# Clinical and Pathological Evidence of Anti-GD2 Immunotherapy Induced Differentiation in Relapsed/Refractory High-Risk Neuroblastoma

**DOI:** 10.3390/cancers13061264

**Published:** 2021-03-12

**Authors:** Jaume Mora, Alicia Castañeda, Maria Cecilia Colombo, Maite Gorostegui, Fernando Gomez, Salvador Mañe, Vicente Santa-Maria, Moira Garraus, Napoleon Macias, Sara Perez-Jaume, Oscar Muñoz, Juan Pablo Muñoz, Ignasi Barber, Mariona Suñol

**Affiliations:** 1 Oncology Department, Pediatric Cancer Center Barcelona, Hospital Sant Joan de Déu, 08950 Barcelona, Spain; acastanedah@hsjdbcn.org (A.C.); mgorostegui@sjdhospitalbarcelona.org (M.G.); vsantamaria@sjdhospitalbarcelona.org (V.S.-M.); mgarraus@sjdhospitalbarcelona.org (M.G.); sperezj@fsjd.org (S.P.-J.); omunoz@fsjd.org (O.M.); jmunoz@sjdhospitalbarcelona.org (J.P.M.); 2 Radiology Department, Pediatric Cancer Center Barcelona, Hospital Sant Joan de Déu, 08950 Barcelona, Spain; mccolombo@sjdhospitalbarcelona.org (M.C.C.); ibarber@sjdhospitalbarcelona.org (I.B.); 3 Interventional Radiology Department, Pediatric Cancer Center Barcelona, Hospital Sant Joan de Déu, 08950 Barcelona, Spain; fgomezm@sjdhospitalbarcelona.org (F.G.); nmacias@sjdhospitalbarcelona.org (N.M.); 4 Nuclear Medicine Department, Pediatric Cancer Center Barcelona, Hospital Sant Joan de Déu, 08950 Barcelona, Spain; Smane@quironsalud.es; 5 Pathology Department, Pediatric Cancer Center Barcelona, Hospital Sant Joan de Déu, 08950 Barcelona, Spain; MSunol@sjdhospitalbarcelona.org

**Keywords:** neuroblastoma, anti-GD2 immunotherapy, naxitamab, differentiation, functional MRI, functional imaging, CHD5

## Abstract

**Simple Summary:**

The anti-tumor activity of anti-GD2 monoclonal antibodies (mAbs) have been demonstrated by the capacity to mediate immunological cytotoxicity but also through direct cell death induction. Recently, studies with anti-GD2 mAbs for high-risk (HR)-neuroblastoma (NB) patients with measurable disease, with or without chemotherapy, have reported significant objective responses. In this subgroup of patients, we observed that, while being treated with the mAb naxitamab, some chemorefractory lesions showed long periods of stable disease. Here, we report a comprehensive imaging evaluation of those lesions correlating with histopathological demonstration of naxitamab-induced tissue differentiation. Our results suggest an undescribed mechanism of action for anti-GD2 mAbs.

**Abstract:**

Background: Neuroblastic tumors (NBTs) originate from a block in the process of differentiation. Histologically, NBTs are classified in neuroblastoma (NB), ganglioneuroblastoma (GNB), and ganglioneuroma (GN). Current therapy for high-risk (HR) NB includes chemotherapy, surgery, radiotherapy, and anti-GD2 monoclonal antibodies (mAbs). Anti-GD2 mAbs induce immunological cytoxicity but also direct cell death. Methods: We report on patients treated with naxitamab for chemorefractory NB showing lesions with long periods of stable disease. Target lesions with persisting ^123^I-Metaiodobenzylguanidine (MIBG) uptake after 4 cycles of immunotherapy were further evaluated by functional Magnetic Resonance Imaging (MRI) and/or Fluorodeoxyglucose (FDG)-positron emission tomography (PET). MIBG avid lesions that became non-restrictive on MRI (apparent diffusion coefficient (ADC) > 1) and/or FDG-PET negative (SUV < 2) were biopsied. Results: Twenty-seven relapse/refractory (R/R) HR-NB patients were enrolled on protocol Ymabs 201. Two (7.5%) of the 27 showed persistent bone lesions on MIBG, ADC high, and/or FDG-PET negative. Forty-four R/R HR-NB patients received chemo-immunotherapy. Twelve (27%) of the 44 developed persistent MIBG+ but FDG-PET- and/or high ADC lesions. Twelve (86%) of the 14 cases identified were successfully biopsied producing 16 evaluable samples. Histology showed ganglioneuroma maturing subtype in 6 (37.5%); ganglioneuroma mature subtype with no neuroblastic component in 4 (25%); differentiating NB with no Schwannian stroma in 5 (31%); and undifferentiated NB without Schwannian stroma in one (6%). Overall, 10 (62.5%) of the 16 specimens were histopathologically fully mature NBTs. Conclusions: Our results disclose an undescribed mechanism of action for naxitamab and highlight the limitations of conventional imaging in the evaluation of anti-GD2 immunotherapy clinical efficacy for HR-NB.

## 1. Introduction

Neuroblastic tumors (NBTs) derive from neural crest cells and are the most common extracranial solid childhood tumors. Neural crest cells delaminate and migrate from the dorsal neural tube and those neuroblastic precursor cells differentiate upon reaching their final embryonic sites into tissues that will constitute the sympathetic nervous system [1]. In vitro and in vivo studies have shown that NBTs originate from a block in the process of normal differentiation [2].

Histologically, NBTs are classified in three categories, neuroblastoma (NB); ganglioneuroblastoma (GNB); and ganglioneuroma (GN). By definition, Schwannian stroma comprises less than 50% of the tumor to be NB. Undifferentiated NB is composed of neuroblastic cells without identifiable neuropil. Tumor cells are small and have no discernible cytoplasm. The nuclei are round, with a salt-and-pepper appearance, and may contain distinct nucleoli. In these undifferentiated tumors, immunohistochemistry shows a pattern compatible with immature ganglionic (neuronal) sympathetic nervous system lineage origin (Phox2b positive). These observations suggest that undifferentiated NB may be locked at an early neuronal differentiation stage, without the capacity to differentiate in response to the differentiating factors driving normal sympathetic neuronal formation. GNB show mature Schwannian stromal component and well-defined microscopic foci of neuroblastic cells in various stages of differentiation. The neuroblastic component of GNB tumors expresses markers of an advanced ganglionic (neuronal) development. GN are composed of mature Schwannian stroma (>90% of the tumor tissue) and scattered neuroblastic cells, which include differentiating neuro-blasts and maturing or mature ganglion cells usually surrounded by satellite cells. Mature Schwann cells represent the dominant component of the tumor, characteristically forming multiple fascicles covered with perineurial cells. GN arises from mature neuronal sympathetic ganglia or adrenal medulla neuronal cells [1]. Differentiated NBTs are associated with good prognosis and lower stage. Therefore, induction of differentiation is seen as therapeutically advantageous and differentiating agents like retinoic acid (RA) have reached the clinical practice [3].

The multi-modal therapeutic approach of high-risk (HR) NB includes chemotherapy, surgery of the primary tumor, and radiotherapy. Treatment drastically reduce the tumor burden in the induction and consolidation phases and can lead to an apparent complete remission (CR) of the disease (referred to as minimal residual disease (MRD)). Most cooperative groups include high-dose chemotherapy with autologous haemopoietic stem cell transplant (ASCT) within the consolidation schema. Patients treated with this standard regimen have >50% recurrence rate with most therapeutic failures occurring during the stage of MRD [4]. After a variable period of undetectable disease, many patients relapse in metastatic foci resistant to cytotoxic therapies, and eventually undergo rapid progression. Immunotherapy has been tested over the last three decades as a potential strategy against MRD. Most clinical experience has focused on mono-clonal antibodies (mAbs) against cell membrane antigens. In 1985, Cheung and colleagues described four mAbs against an unknown glycolipid antigen on the surface of human NB cells: GD2 [5]. Disialoganglioside GD2 is a sialic acid-containing glycosphingolipid expressed on the cell surface membrane playing an important role in cell-to-cell communication and the attachment capacity of cells [6]. In normal human tissues, GD2 expression is restricted to neurons, skin melanocytes, and unmyelinated type C nerve fibers—mostly the vagus [7].

Innate anti-tumor immunity against NB has been hypothesized because some NB can spontaneously regress [8]. However, an active adaptive immunity against NB has been difficult to demonstrate in HR patients. The large tumor bulk and the rapid proliferation overwhelm the immune system. Additionally, the low number of somatic mutations makes NB poorly immunogenic, and NB has developed a sophisticated immunosuppressive microenvironment to ensure that T-cell immunity cannot become efficient [9]. The anti-GD2 mAbs anti-tumor efficacy in vivo has been demonstrated by the capacity of post infusion sera to mediate complement-dependent cytotoxicity (CDC) and antibody-dependent cellular cytoxicity (ADCC). The ADCC property of anti-GD2 mAbs is most efficient when effector cells like natural killer (NK), granulocytes, and macrophages are potentiated by cytokines [10]. Sargramostim (GM-CSF) has shown both in vitro and in vivo to enhance ADCC through direct activation of monocytes, macrophages, dendritic cells, and indirect T-cell activation via tumor necrosis factor (TNF), interferon, and IL-1 [11].

Other strategies to improve antibody immunotherapy in NB include the use of stimulators of the innate immune system. β-glucans, glucose polymers that can induce TNF secretion and ADCC by NK cells, monocytes, and neutrophils, have been tested in combination with anti-GD2 mAbs [12]. Another reported strategy attempts to sensitize tumor cells to ADCC and CDC with nontoxic concentrations of fenretinide [13]. Finally, studies have shown that mAbs targeting GD2 inhibit tumor cell growth not only by ADCC and CDC, but also directly through cell death induction [14]. The mouse mAb 14G2a showed dose-dependent decrease of survival of IMR-32 NB cells by inducing cell death through an apoptosis mechanism [15].

Anti-GD2 mAbs were initially approved to treat MRD. Most recently, studies with anti-GD2 mAbs have been performed in HR-NB patients with refractory/resistant soft tissue or bone/bone marrow (B/BM) disease. We and others have reported significant objective responses in both groups showing significant improvements in the outcome of patients with HR-NB. A report from Childrens Oncology Group (COG) of a cohort of patients with relapsed/refractory NB treated with irinotecan (I), temozolomide (T), dinutuximab, and GM-CSF showed major objective responses in 22 (41.5%) of 53 patients [16]. Interestingly, this study describes the same number of patients achieving stable disease (SD), 22 of 53. In our experience with the combination of naxitamab, I/T, and GM-CSF, for patients with evaluable or measurable chemo resistant disease, objective responses were documented in 17 (47%) of 36 evaluable patients [17]. Similarly, 10 (27%) of the 36 achieved SD. We became intrigued in the subgroup of lesions that, while on naxitamab treatment, showed long periods of stability, characterized by persistent MIBG uptakes in asymptomatic patients. This subgroup of lesions was further investigated with functional MRI and FDG-PET in order to query the persistent MIBG avid lesions for signs of differentiation/maturation. Given the limitations encountered to ascertain the status of the persistent MIBG avid lesions by functional imaging, we prospectively performed biopsies of those lesions. In this study, we present evidence of naxitamab-induced differentiation of refractory HR-NB.

## 2. Materials and Methods

This study was performed in agreement with the declaration of Helsinki on the use of human material for research. In accordance with the ethics committee of Hospital Sant Joan de Déu (HSJD), written informed consent of all patients or their custodians was obtained for “scientific use of tumor tissue not needed for histopathological diagnosis” in the admission contract of HSJD (M.1608-C).

We report on patients treated at HSJD with naxitamab-based immunotherapy for chemo refractory NB. Eligibility criteria included patients with HR-NB (stage M at age >18 months or MYCN-amplified stages L1-2 at any age) with refractory disease documented following ASCT or induction regimens including chemotherapy and surgery. Primary refractory disease is defined as an incomplete response (persistent detectable disease) in the bone and/or BM, or soft tissues, to chemotherapy induction regimens that include at least 5 cycles of chemotherapeutic agents including alkylators and platinum-containing compounds. Patients with a history of previous relapse and subsequent incomplete response to rescue regimens were entered as secondary (or further) refractory. Patients were enrolled on the Ymabs protocol 201 (EudraCT 017-001829-40) with naxitamab and GM-CSF for primary refractory patients in the bone and/or BM (*n* = 27); or the combination of naxitamab, irinotecan, temozolomide, and GM-CSF (hu3F8 or naxitamab, Irinotecan, and Temozolomide = regimen HITS) for refractory HR-NB (*n* = 44) through compassionate use.

Patients were eligible for immunotherapy if major organ toxicity was grade <2 by Common Terminology Criteria for Adverse Events Version 4.0.

### 2.1. Immunotherapy Treatment

Naxitamab-based immunotherapy cycles comprised priming doses of subcutaneous GM-CSF for 5 days at 250 μg/m^2^/day (days −4 to 0), followed by naxitamab + subcutaneous GM-CSF for 5 days at 500 μg/m^2^/day (days 1–5). Naxitamab was infused intravenous over 30 min, at 3 mg/kg/day on days 1, 3, and 5 for a total dose of 9 mg/kg per cycle. GM-CSF was not given if the ANC was >20,000/μL. Treatment cycles were repeated every 4 weeks (±1 week) for a total of 5 cycles or until CR followed by 5 additional cycles every 4 weeks (±1 week).

HITS cycles comprised irinotecan 50 mg/m^2^/day intravenously plus temozolomide 150 mg/m^2^/day intravenous or oral (days 1–5); naxitamab 2.25 mg/kg/day intravenous over 30 min, days 2, 4, 8, and 10 (total 9 mg/kg or 270 mg/m^2^ per cycle), and GM-CSF 250 mg/m^2^/day subcutaneously, days 6–10, as previously reported (18).

Naxitamab treatment was outpatient in all cases.

### 2.2. Other Treatments

All patients received daily oral supplementation of docosahexaenoic acid triglyceride (DHA-TG) at 0.25 g/kg, half administered in a single oral intake and the rest in the other two administrations during the day, matched with meals.

None of the patients received cis-retinoic acid.

### 2.3. Disease Evaluation

Disease status was assessed at study entry by histology of BM biopsies/aspirates obtained from bilateral posterior and bilateral anterior iliac crests, 123I-MIBG SPECT scan, and whole body MRI. FDG-PET was used for MIBG non-avid cases at diagnosis. Four BM aspirates and 123I-MIBG SPECT scan or FDG-PET scans were performed every 2 cycles in all patients to assess response. Quantitative reverse transcription-polymerase chain reaction was used to assess MRD, as described [18], in pooled heparinized BM aspirates before treatment and after every two cycles of immunotherapy. Disease response was defined according to the international neuroblastoma revised criteria [19].

Treatment could be continued for a response of SD or better, provided that patients remained clinically asymptomatic and had adequate tolerance to treatment. Target lesions showing persisting 123I-MIBG SPECT scan uptake after 4 cycles of immunotherapy were further evaluated by functional MRI and/or FDG-PET. 123I-MIBG SPECT scan avid lesions that became non-restrictive on apparent diffusion coefficient (ADC) and/or FDG-PET negative (SUV < 2) were planned for percutaneous biopsy.

### 2.4. Functional MRI

Diffusion-weighted (DW) Magnetic Resonance Imaging (MRI) provides “functional” information regarding the free diffusivity of water molecules. The restriction of water diffusion can be quantitatively analyzed with the calculation of the ADC. It has been proved that highly dense cellular areas are related to restricted diffusion and low ADC values in comparison to areas with less cellular density that show higher ADC values [20]. Recently, we reported that MRI is useful for the detection of bone involvement in NB and that quantitative DWI is able to differentiate low cell density skeletal lesions (which might be considered as non-active or residual) from highly cellular lesions (associated with tumor viability) [21].

Whole-body MRI was performed with a 3 Tesla MR (Philips Ingenia) using specific head and body surface coils. Each study included coronal T1 weighted images (multipoint DIXON), coronal T2 weighted STIR images, abdomen and pelvis transverse T2 fat-suppressed weighted SPAIR images, and transverse whole-body DWI with b values of 0–1000 mm^2^/s. The ADC and exponential ADC images were automatically generated from both b values on the operating console. MR images were reviewed by two experienced pediatric radiologists (M.C.C. and I.B.). SPECT MIBG images were reviewed independently by nuclear medicine specialist (S.M.). The maximum interval between both studies was 7 days.

MRI criteria for bone lesions were: T1 hypointense and STIR T2 hyperintense focal bone lesion. Ill-defined or diffuse bi-lateral and symmetric high T2 signal and low T1 signal bone marrow areas were not considered as bone lesions to exclude normal hematopoietic bone marrow. Quantitative ADC values of the bone lesions were obtained using Impax viewer software (AGFA) by using the minimum diameter region of interest (ROI; 4.2 mm), corresponding to a 13.85 mm^2^ area.

**Figure 1:**
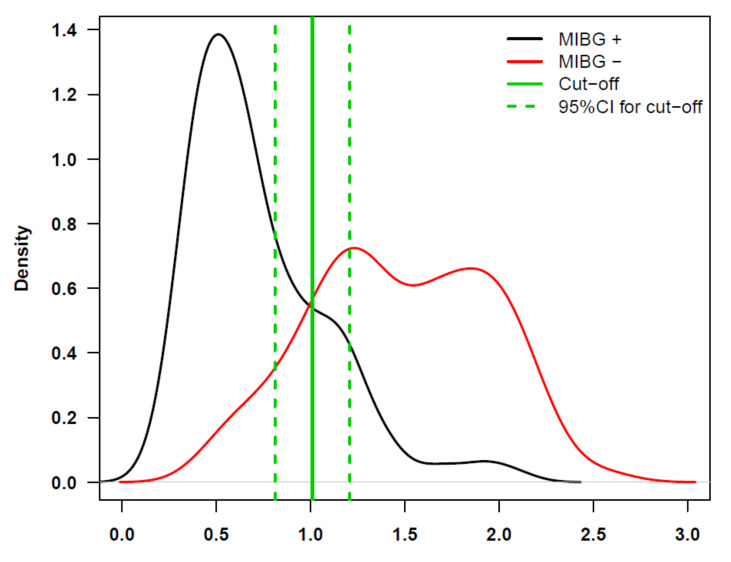
ADC in MRI+ observations.

In order to estimate an optimal cutoff for ADC, that best discriminates positive versus negative MIBG findings, we used the Youden index maximization method [22]. We analyzed the data values of 348 MRI+ observations from 44 HR-NB patients studied by MRI and MIBG. Bootstrap was used to determine the confidence intervals (CI) [23]. The cutoff obtained was 1.01, 95% CI = (0.81, 1.21). The density plot with the cutoff and CI is shown in Figure 1.

### 2.5. Biopsy Procedure

Data from 123I-MIBG/FDG-PET and MRI DWI were carefully evaluated to select target lesion. MIBG avid lesions with high ADC and/or FDG-PET negative (SUV < 2) were chosen. Previous MRI and anatomical landmarks were used for procedural planning. For biopsy planning, a cone beam computed tomography (CBCT) was performed on an Allura Xper FD20/20 (Philips, Best, The Netherlands) system. Entry and target points were defined using a dedicated guidance software (XperGuide). Needle trajectory was corrected to achieve the safest path for the biopsy, avoiding critical structures such as vessels or nerves. For target planning, the CBCT images and former restrictive MRI were fused. Anatomical landmarks and 123I-MIBG/FDG-PET scans were used to corroborate the target lesion.

Under general anesthesia, patients laid on the angiography suit table choosing the best position for the predicted route of the biopsy. The CBCT was acquired (XPerCT, Philips, Best, The Netherlands) using initially the high dose 10 s protocol. After, image overlay was performed on the workstation and according to the merged images target and entry point were defined. Anatomical landmarks were used to confirm the 123I-MIBG/FDG-PET images and real time fluoroscopy 3D image guidance software (XperGuide Philips, Best, The Netherlands) was applied for needle positioning. Either 10 Ga Arrow OnControl^®^ (Teleflex, Morrisville, NC, USA) or 14 Ga Bonopty^®^ (Apriomed, Upsala, Sweden) were employed to obtain the histology sample. Entry point and needle trajectory monitored the needle path and once in position a new low dose (with superior and inferior collimation) 6 s protocol CBCT and image overlay was performed to confirm the right biopsy needle location adjusting the window width and level to minimize metal artifacts. We aimed at obtaining samples from different areas by angulating the co-access needle. Finally, after samples were removed, the co-access needle was retrieved and low dose 6 s CBCT was acquired to rule out complications.

### 2.6. Immunohistochemistry

Immunohistochemical (IHC) analysis was performed on formalin-fixed, paraffin-embedded (FFPE) tissues as previously described (34) using rabbit-polyclonal anti-CHD5 antibody (Strategic Diagnostics, DE, USA) at a 1:1000 dilution for 1 h; mouse-polyclonal anti-neurofilament protein, 68 kD (NF68) antibody (Invitrogen. Waltham, Massachusetts. USA) 1:300 dilution, 1 h; mouse-polyclonal anti-Glial fibrillary acidic protein (GFAP) antibody (Novocastra, UK) 1:200 dilution, 2 min; rabbit-polyclonal S-100 protein antibody (Diagnostic Biosystems. Pleasanton, California. USA) 1:800 dilution, 15 min. GD2 analysis was performed by IHC analysis on frozen tissues using mouse anti-human GD2 antibody (BD Pharmingen. San Diego, California. USA) 1:300 dilution, 15 min.

All slides were examined by pediatric pathologist (M.S.) using an Olympus BX41 light microscopy. Assessing staining and score of both percentage of positive cells and staining intensity were done as follows: 0, negative; 1, weak; 2, strong; and 3, very intense staining. Integer values were assigned to the proportion of positive cells: 25% = 1; 25–75% = 2; >75% = 3. For CHD5, intensity and positive cell values were multiplied to provide a single score for each case.

## 3. Results

Since March 2018, twenty-seven (17 primary and 10 secondary, refractory) patients were enrolled on protocol Ymabs 201 (Ymabs Therapeutics EudraCT 017-001829-40). Patients enrolled in this trial had refractory disease exclusively in the bone/bone marrow (BM) compartment by MIBG and/or conventional cytomorphology examination of the BM. Over the course of immunotherapy, two (7.5%) of the 27 patients showed persistent, stable, bone lesions on MIBG that turned ADC high and/or PET-FDG negative. These lesions were biopsied before any other intervening treatments and are included in this report (Table 1, pts #1 and #2). Seven more patients (Table 1, pts #3–8 and 14) received immunotherapy according to the 201 protocol but also received HITS cycles because of relapse or persistent/refractory disease FDG-PET positive and/or DWI with low ADC values.

Since November 2017, forty-four (16 primary and 28 secondary) chemo and/or anti-GD2 resistant patients received chemo/immunotherapy HITS cycles (17). All patients had refractory, non-progressing disease after at least 2 cycles of chemo-radiation before receiving HITS cycles. Upon treatment initiation, all evaluable lesions were MIBG positive and ADC low or FDG-PET positive. Twelve (27%) of the 44 patients developed persistent MIBG positive but FDG-PET negative and/or ADC high lesions while receiving HITS cycles. As described above, 7 of these 12 patients had received naxitamab and GM-CSF prior.

A flow chart summary of all patients managed with naxitamab at HSJD is shown in Supplementary Materials. This chart describes the outcome and the origin of the 14 patients with persistent MIBG+/FDG-PET^-^ target lesions that were percutaneously biopsied and are the subjects of this study. The results from biopsy specimens obtained from those 14 patients are summarized in Table 2.

### 3.1. Functional Imaging

**Figure 2:**
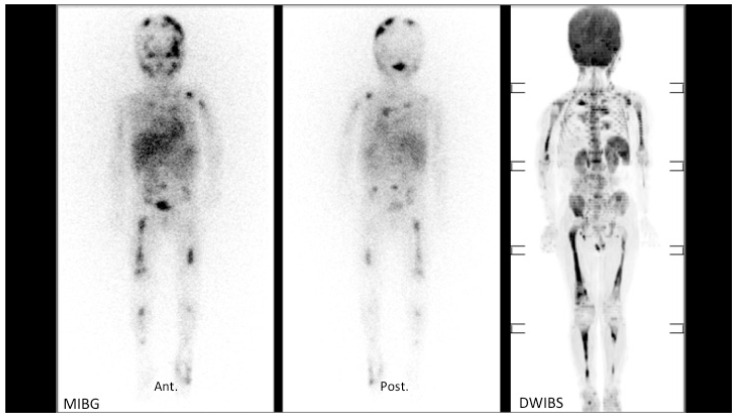
Correlation example of positive bone lesions by ^123^I-Metaiodobenzylguanidine (MIBG) and diffusion-weighted (DW) Magnetic Resonance Imaging (MRI) for patient #9 (Table 1) before chemo-immunotherapy treatment.

Figure 2 shows an example of a good correlation between ^123^I-MIBG SPECT scan bone uptake and areas of restricted diffusion on a Coronal 3D MIP reconstructed DWI-MR imaging (DWIBS) for patient #9 (Table 1). These studies were performed before treatment with naxitamab-based chemo-immunotherapy.

Left (anterior view) and middle (posterior view) panels: positive MIBG in multiple skeleton bones. Right panel: areas of restricted diffusion on a Coronal 3D reconstructed DW-MRI imaging (DWIBS) of the same patient. Note the limitation of skull bone involvement evaluation on DWIBS compared to MIBG.

**Figure 3:**
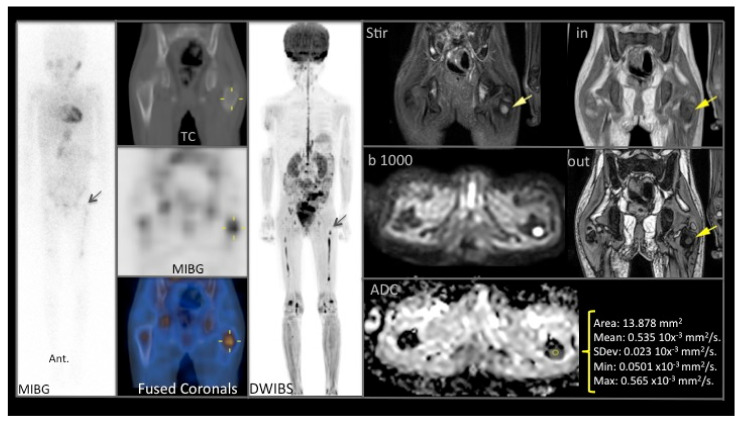
Example of a good correlation between proximal left femoral metaphysis ^123^I-Metaiodobenzylguanidine (MIBG) bone uptake (left column) and MRI/DW (middle and right panels) for patient #13 (Table 1), showing T1 hypointense, STIR T2 hyperintense focal bone lesion with restricted diffusion (b 1000), and low ADC (<1.01) values.

Figure 3 shows the chemorefractory femur lesion before naxitamab-based chemo-immunotherapy for patient #13 (Table 1). After 8 HITS cycles, the persistent MIBG^+^ lesion was biopsied showing GN (see below).

**Figure 4:**
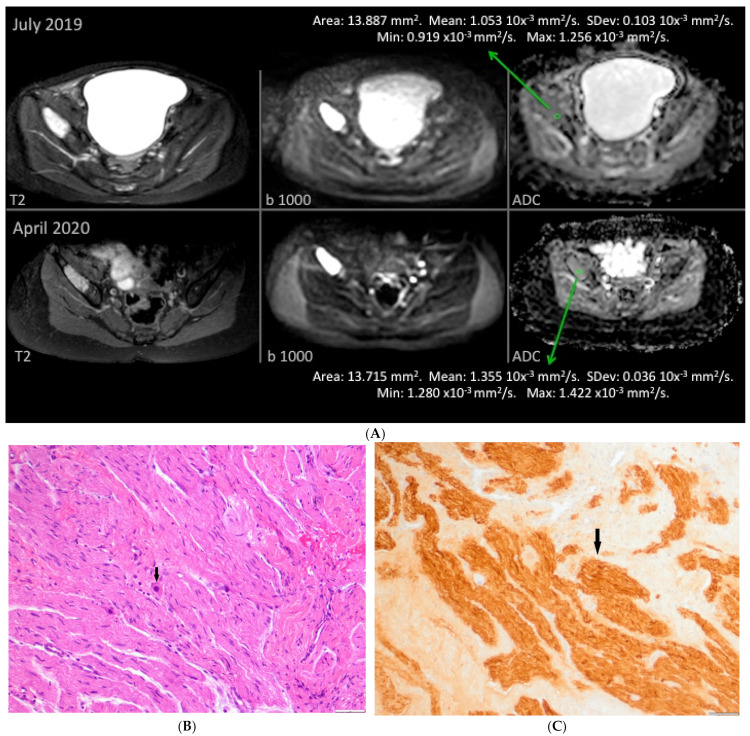
Correlation of functional imaging for patient #1 (Table 1) and histopathology. (**A**) Correlation between pre-immunotherapy treatment (top panels) functional imaging of chemo refractory iliac bone lesion and post immunotherapy evaluation (bottom panels: STIR T2 hyperintense focal iliac bone lesion with restricted diffusion -b 1000- and high- > 1.01- ADC values) of the same iliac lesion. (**B**) Bone tumor from patient #1 post immunotherapy. This sample shows ganglioneuromatous tissue with Schwannian stroma and some mature ganglion cells (arrow). This sample corresponds to the iliac bone lesion described by functional imaging in (**A**) (bottom panels). (**C**) S100 immunostaining highlighting the Schwannian stroma (arrow) of the same iliac bone tumor.

Patient #1 (Table 1) received naxitamab and GM-CSF for primary refractory neuroblastoma in multiple bones. After 4 cycles only, one iliac bone lesion persisted positive on MIBG. PET-FDG was negative and DWI with high ADC is shown in Figure 4A.

### 3.2. Pathology

Twelve (86%) of the 14 cases with persistent, stable, MIBG+/FDG-PET- lesions were successfully biopsied. In total, 16 (patient #8 had 2, #11 had 3, and #12 had 2 independent specimens) percutaneous tissue samples with representative bone or soft tissue tumor were obtained. The histological picture was compatible with ganglioneuroma maturing subtype in 6 (37.5%); ganglioneuroma mature subtype with no neuroblastic component in 4 (25%); differentiating neuroblastoma with no schwannian stroma in 5 (31%); and undifferentiated neuroblastoma without schwannian stroma in one (6%). Overall, 10 (62.5%) of the 16 specimens are histopathologically fully mature ganglioneuromatous tissues, whereas 6 (37.5%) with same imaging findings (123I-MIBG SPECT scan positive, FDG-PET negative, high ADC) consisted of neuroblastoma. Summary of all histopathology findings can be found in Table 2.

A histological picture of ganglioneuroma in the bone from patient #1 is shown in Figure 4B and Figure 3C corresponding to the iliac bone lesion described by functional imaging in Figure 4A. In Supplementary Materials, the histological picture of the original undifferentiated NB of this patient is shown.

**Figure 5:**
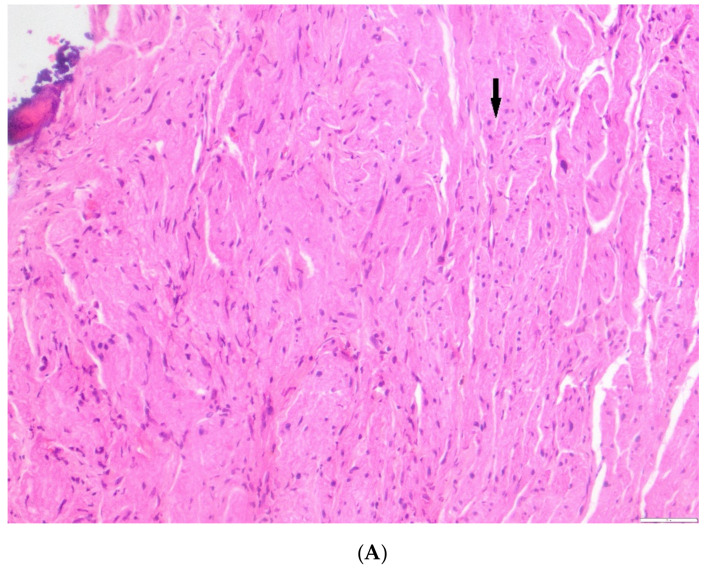

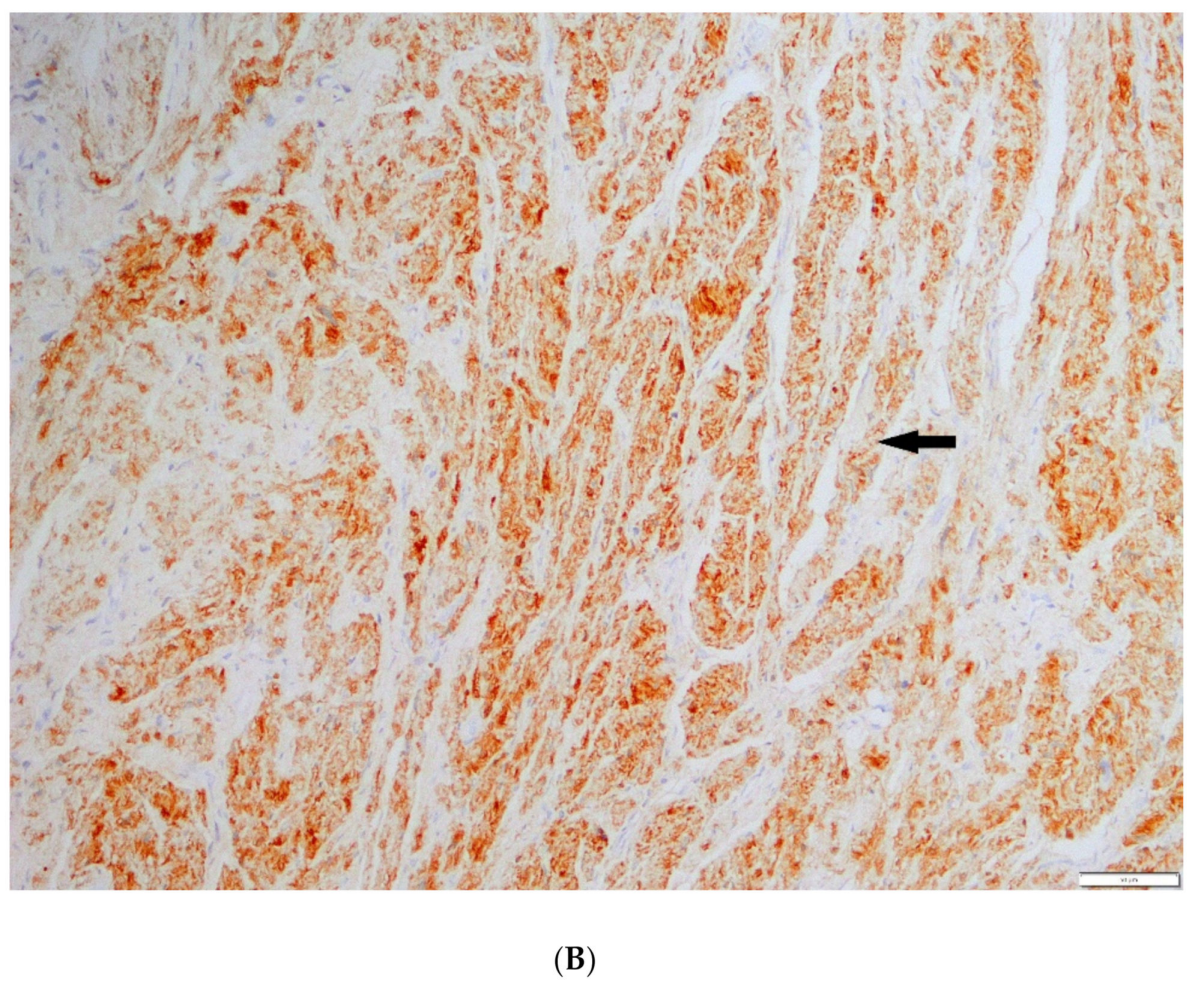
Describes case #4 (Table 1 and Table 2), a patient managed initially with naxitamab and GM-CSF with no objective response (MIBG and PET-FDG diffusely positive in multiple bone lesions). Subsequently patient received HITS showing an objective response (lower Curie score) and PET-FDG turning negative. (**A**) Bone tissue diffusely infiltrated by tumor composed of Schwannian stroma (arrow), (**B**) S-100 immunostaining depicting massive Schwannian stroma (arrow).

Figure 5 summarizes case #4 (Table 1 and Table 2) with bone tissue diffusely infiltrated by Schwannian stroma (Figure 5A) in a patient managed initially with naxitamab and GM-CSF with lack of objective response (123I-MIBG SPECT scan and FDG-PET diffusely positive in multiple bone lesions). Subsequently, patient received chemo-immunotherapy HITS showing an objective radiological response (lower Curie score) and FDG-PET turning negative. S-100 immunostaining depicting massive Schwannian stroma defining the histological picture of ganglioneuroma in the bone specimen for patient #4 (Table 1) is shown in Figure 5B.

**Figure 6:**
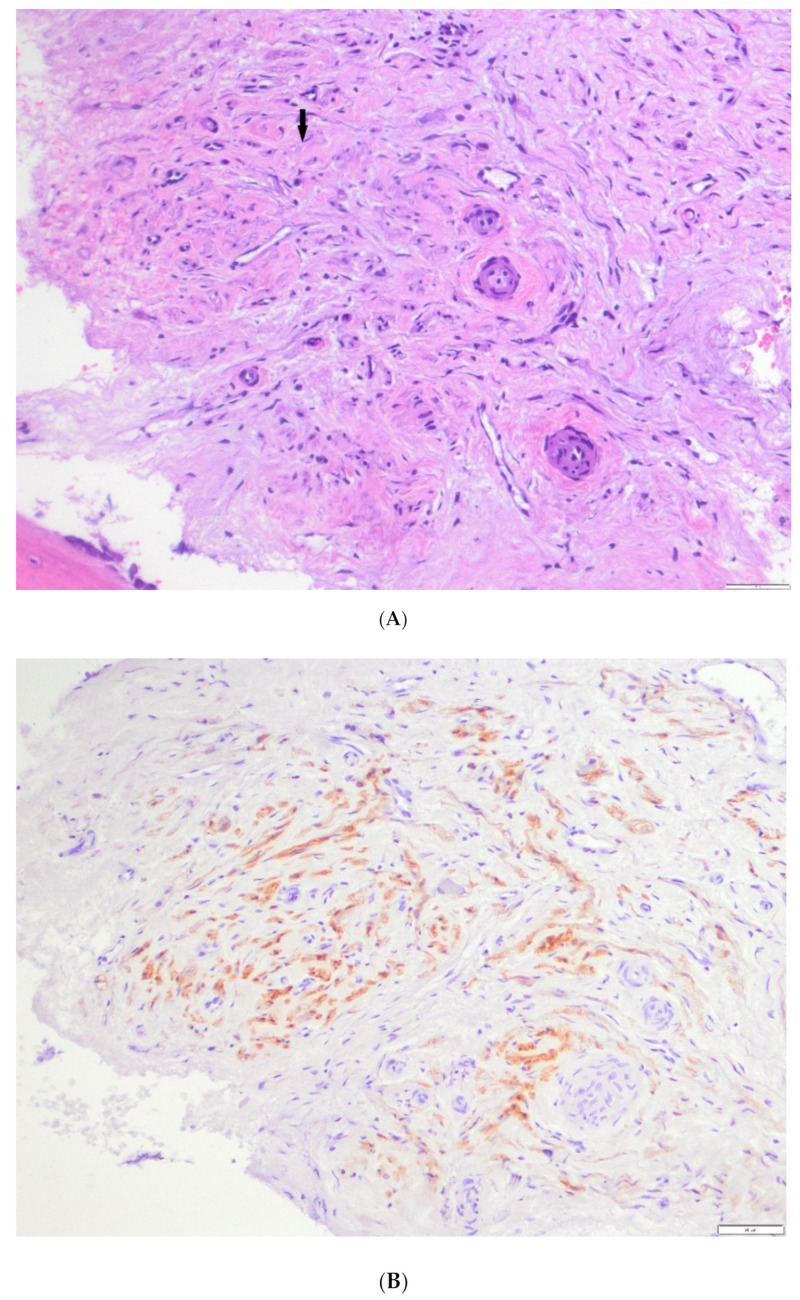
Describes case #5 (Table 1 and Table 2) biopsy of the right sphenoid bone. (**A**) Histology consists of only Schwann tissue (arrow) with no evidence of neuroblastic cells. (**B**) Schwannian stroma shown by S100 immunostaining.

Figure 6 describes case #5 (Table 1 and Table 2). Patient was diagnosed of stage 4 NB presenting with sudden onset of amaurosis due to a rapidly growing NB invading the sphenoid. After 7 cycles of induction chemotherapy, 123I-MIBG SPECT scan persisted positive in the sphenoid and clivus (Curie score = 1). The patient then was enrolled in the 201 Ymabs Trial and after 4 cycles 123I-MIBG SPECT scan became negative. The patient received 4 more cycles of immunotherapy and after the 8th cycle, 123I-MIBG SPECT scan became positive again in the right side of the sphenoid. Because of this abnormal uptake in the ^123^I-MIBG SPECT scan interpreted as NB recurrence, the patient was taken off trial and received 2 cycles of irinotecan and TMZ, and radiotherapy to the skull base. Post radiotherapy, 123I-MIBG SPECT scan persisted positive in the sphenoid area with no other sites of progression of disease. FDG-PET was positive suggestive of active disease. The patient then received chemo-immunotherapy according to the HITS protocol and after 4 cycles ^123^I-MIBG SPECT scan remained positive but FDG-PET became negative. Eventually, biopsy of the right sphenoid was undertaken and histology is shown in Figure 6A consisting of only Schwann cells. Schwannian stroma is highlighted by S100 immunostaining (Figure 6B) with no evidence of neuroblastic component in the sample.

## 4. Discussion

We here report evidence of anti-GD2 mAb naxitamab-based immunotherapy-induced differentiation of undifferentiated, high-risk, refractory neuroblastoma. The observations made by functional imaging suggested mature, metabolic non-active tissue, for lesions persistently MIBG positive. The histological confirmation of terminally differentiated neuroblastic tissue in 62.5% of specimens obtained after naxitamab only or in combination with I/T, suggests that naxitamab may act as a differentiating agent. The anti-tumor activity of anti-GD2 antibodies is well described with all mechanisms reported leading to cell death. Indeed, cell death was induced in all these cases showing complete remission of lesions concomitant to those that persisted. For those persistent sites, according to our results, an alternative mechanism of action for naxitamab, i.e., induction of differentiation, was elicited. The concomitance of different mechanisms of action for naxitamab in the same patient suggests that tissue microenvironment and/or local characteristics of the immunological response may determine how tumor cells react to anti-GD2 mAbs. Indeed, chemoimmunotherapy was introduced with the intention to change the microenvironment, modify vascularity, to create neoantigens, and to induce immunogenic cell death [16].

Neuroblastoma cells have the capacity to differentiate when triggered by various agents. In 1927, Cushing and Wolbach described a patient with GN in a lymph node [24]. Since GN do not metastasize, they assumed that the tumor cells in the lymph node derived from prior NB cells, suggesting that NB cells could spontaneously differentiate. The report by Cushing and Wolbach sustained clinical relevance of the laboratory observations of NB cells maturing in culture. The first demonstration of in vitro differentiation of a NB cell line was reported in 1981 by S. Pahlman and colleagues from Lund University. The human SH-SY5Y NB cells (a subclone of the Sloan-Kettering SK-N-SH cell line) were shown to differentiate morphologically and biochemically in response to phorbol esters [25]. The induction of neurites in these cells occurred along with an increase of norepinephrine and neuron specific enolase. Since that first report, a number of differentiation protocols have been published. Retinoids and growth factors such as nerve growth factor were readily identified among potent differentiation factors of NB cells. These laboratory observations generated the idea that patients with NB might be treated by differentiating agents.

One of the most potent differentiation inducers of NB in vitro is retinoic acid (RA). Treatment of NB cell lines with all-trans-retinoic acid (ATRA) causes an arrest of cell proliferation. In the mid-1980s, laboratory experiments showed that 13-cis-retinoic acid (13-cis-RA) induced differentiation of promyelocytic leukemia and were used in human trials with some objective responses. Using an intermittent schedule, 13-cis-RA was dose-escalated to a maximally tolerated dose of 160 mg/m^2^/day in post-transplant NB patients. In the CCG-3891, phase 3 trial patients were randomized to consolidation therapy with no further therapy or 13-cis-RA at 160 mg/m^2^/day bid for 2 weeks each month over a 6 months period [26]. A survival benefit was reported for patients receiving 13-cis-RA at the MRD stage. Over the years, however, the survival benefit reported in the original article was amended and overall survival was not significantly improved by cis-RA [27].

Targeted immunotherapy using anti-GD2 monoclonal antibodies represents an important clinical advance in the treatment of HR-NB. In a landmark study published in 2010 by the cooperative North American group COG, the addition of the anti-GD2 monoclonal antibody ch14.18 (dinutuximab) combined with cytokines and 13-cis-RA improved the survival rates compared with 13-cis-RA alone in the post-consolidation phase [28]. As a consequence of this and subsequent studies, in 2015, dinutuximab was approved in Europe and the US for the treatment of HR-NB, and is now considered part of the standard of care [29].

Anti-GD2 mAbs have three well-proven mechanisms of action (MOA) against GD2-expressing tumor cells: (1) induction of phagocytosis by macrophages and destruction of tumor cells by natural killer (NK) cells and granulocytes via ADCC; (2) lysis of tumor cells via CDC; and (3) direct induction of cell death due to specific binding to GD2. In ADCC anti-GD2 mAbs engage Fcγ receptors on the surface of NK cells and granulocytes, followed by the release of cytotoxic granules (serine proteases and perforin, a glycoprotein that creates pores in cell membranes [30]), causing Fc dependent phagocytosis and lysis of tumor cells [31]. CDC is induced through binding of the serine protease complex C1 to the Fc domains of mAbs binding to antigens expressed on tumor cells [32]. This classic complement pathway results in activation of a cascade signaling causing the membrane attack complex to disrupt the target cell. Anti-GD2 mAbs also may induce cell death directly without involving immune mechanisms, combining features of apoptosis and necrosis in GD2-positive tumor cell lines but not in GD2-negative tumors [33]. Direct induction of mAb-mediated tumor cell death occurs in a dose-dependent manner, with the strongest cytotoxic effects observed in tumor cells with the highest expression of GD2. In addition, anti-GD2 mAbs inhibit attachment of circulating malignant cells to protein components of the extracellular matrix [34], potentially representing a fourth MOA. Finally, several experimental data demonstrated that autophagy is induced in response to the 14G2a mAb treatment in the IMR-32 cell line [35]. In this study, we show evidence of neuroblastic maturation in response to either naxitamab and GM-CSF only or in combination with I/T chemotherapy in some chemo refractory loci of disease concomitant with immunological cytotoxicity occurring in other sites. The mechanisms involved in this unusual response are currently being investigated.

Undifferentiated NB occasionally exhibit neuroblastic maturation in response to chemotherapy. Previously, we reported the assessment of CHD5 gene and protein expression in post-therapy specimens of NB showing that tumors with evident neuroblastic maturation displayed both CHD5 gene and protein reactivation [36]. Interestingly, Higashi M et al. reported that treatment with 13-cis-RA induces neuronal differentiation only in NB cells that upregulate CHD5 [37]. In their in vitro study, they showed that CHD5 expression is crucial for neuronal differentiation induced by either 13-cis-RA or TrkA/NGF signaling. In NBTs, CHD5 is essentially expressed in the nucleus of differentiating neuroblastic cells and ganglion cells, and absent in the Schwannian stromal component. In NB, CHD5 nuclear staining was strongly associated with established favorable prognostic variables like low clinical stage, age at diagnosis <12 months, and favorable histology [36]. We had shown statistically significant association between high CHD5 immunoreactivity and favorable outcome [36]. In this study, we show increased expression of CHD5 in those cases with histology compatible with differentiating NB (Table 2). The possibility that anti-GD2 mAbs might induce CHD5 expression as the mechanism to explain neuronal differentiation is currently being explored. Additionally, the implications on survival of anti-GD2-induced differentiation is unclear at this time. All patients with mature NBTs in this study were stopped treatment and long-term follow-up is required to demonstrate whether recurrences might occur on those induced mature sites of disease.

Functional imaging evaluation in NB is becoming more sophisticated and interpretation of results in the context of new therapies is not simple. In this study, we compared ^123^I-MIBG SPECT scans, FDG-PET scans, and functional imaging with DWI MR. Several papers have demonstrated the value of DWI MR in the evaluation of skeletal metastasis in other malignancies, however, the evaluation of bone involvement in NB has been considered a drawback for MRI with limitations in the capacity to differentiate between viable tumor and non-viable residual lesion. Previous results of our group [21] suggest that active metastatic bone lesions shown by MIBG are also evident on MRI and show restricted diffusion compared to “residual” bone lesions that are not FDG-PET avid. Complementary use of whole body MRI DWI and MIBG scintigraphy increases diagnostic accuracy in the evaluation of bone involvement. Furthermore, sophisticated techniques for percutaneous biopsy like cone beam computed tomography (CBCT) or robot-assisted techniques recently implemented permit easier and safer access to tissue samples even in difficult bone sites allowing a definitive assessment of the disease status in response to targeted therapies like anti-GD2 mAbs. Our study highlights the significant limitations of each of the imaging techniques when used in isolation (like ^123^I-MIBG SPECT) for the evaluation of response. These results can help guide the implementation of more accurate criteria for evaluating NB response in clinical trials where anti-GD2 immunotherapy is involved.

## 5. Conclusions

Patients with refractory HR-NB treated with naxitamab-based immunotherapy display a variety of responses ranging from immunological-induced cytotoxicity and complete disappearance of imaging detectable lesions; progressive lesions with increased size and activity in functional imaging; and stable, persistent 123I-MIBG positive but FDG-PET negative lesions. We biopsied a series of cases with persistent, stable, asymptomatic lesions after at least 4 cycles of naxitamab-based immunotherapy showing that two thirds of them represent fully mature neuroblastic tissues. These results suggest an alternative, yet undescribed, mechanism of action of anti-GD2 mAbs, and highlight the limitations of current functional imaging to evaluate the anti-tumor effects of HR-NB treated with anti-GD2 antibodies.

## Figures and Tables

**Table 1:** Clinical summary of the 14 patients included in the study.

	Stage	MYCN	Diagnosis	Induction	Disease Status	DHA	Immunotherapy	hu3F8/HITS Cycles	Chemo/RDT	Best Response	Target Sites	Status	F/U
1	M	NA	10/01/2018	CCCG NB2014	PR	Y	10/14/2019	7/	No	SD	Iliac bone	A	14
2	M	NA	11/07/2017	CCCG NB2014	PR	Y	11/18/2019	7/	No	SD	L3 vertebral body	A	13
3	M	NA	08/01/2018	CCCG NB2014	PR	Y	03/11/2019	7/7	Y	SD	Left tibia	A	21
4	M	NA	02/01/2014	COJEC/CTV ×2	SR	Y	03/25/2019	6/4	Y	SD	Right Femur	A	21
5	M	NA	12/01/2018	N7	PR	Y	07/08/2019	7/2	Y	SD	Sphenoid bone	A	17
6	M	NA	02/01/2018	CCCG NB2014/IT × 2	SR	Y	05/13/2019	2/11	y	PR	T4 vertebral body	A	19
7	M	NA	05/01/2015	GPOH NB2004/IT × 2	SR	Y	09/17/2018	6/10	Y	SD	Right Iliac bone	A	27
8	M	NA	03/01/2017	GPOH NB2004/IT × 2	SR	Y	01/27/2020	2/4	Y	PR	T7 vertebral body and left iliac	A	11
9	M	NA	09/01/2018	GPOH NB2004	PR	Y	10/08/2019	/7	Y	SD	Skull/Epidural Soft tissue	A	14
10	M	NA	11/01/2017	N7	PR	Y	11/20/2018	/10	Y	SD	3 Bones/	A	25
11	M	NA	07/01/2018	China BCH-2007	PR	Y	02/19/2019	/17	Y	PR	Left Iliac Bone/Femur/Sacrum	A	22
12	M	NA	07/01/2012	COG ANBL0531	PR	Y	12/17/2019	/6	Y	SD	Paravertebral soft tissue	A	12
13	M	NA	10/23/2018	CCCG NB2014	PR	Y	07/30/2019	/8	Y	PR	Left Femur	A	17
14	M	NA	11/04/2018	COG ANBL0531/IT×2	SR	Y	06/12/2019	5/9	Y	SD	Mandible	A	18

Notes: INRG stage; diagnosis in mm/dd/yyyy; induction regimens used prior to naxitamab treatments: CCG = Chinese cancer group; CTV = cyclophopsphamide/topotecan; IT = irinotecan/TMZ; disease status at initiation of naxitamab therapy; DHA = docosahexaenoic acid triglyceride treatment; start date of naxitamab immunotherapy in mm/dd/yyyy; chemo/RDT: chemotherapy or radiation as rescue; INRC criteria for best response assessment: SD = stable disease; PR = partial response; status A = alive; F/U: follow-up in months.

**Table 2:** Histopathological findings of all cases in the study.

	Histopathology	CHD5
1	Ganglioneuroma	<10%
2	Differentiating Neuroblastoma	40%
3	Schwannian Stroma	Only Schwann cells
4	Ganglioneuroma	Only Schwann cells
5	Schwannian Stroma	Only Schwann cells
6	Undifferentiated NB	Negative
7	Differentiating Neuroblastoma	Few neuroblasts
8	Schwannian Stroma/Differentiating NB	Only Schwann cells/
9	Differentiating Neuroblastoma	60%
10	NA	NA
11	Ganglioneuroma x3	>80%
12	SR Differentiating Neuroblastoma x2	90%
13	Ganglioneuroma	Decalcified Tissue. NE
14	NA	NA

Notes: NA = not available; SR = Schwannian stroma rich; NE: non evaluable.

## Data Availability

Data is contained within the article or Supplementary Material.

## Supplementary Materials

The following are available online at https://www.mdpi.com/2072-6694/13/6/1264/s1, Figure S1: Histological picture of undifferentiated NB from patient #1 at diagnosis (before immunotherapy). A flow chart summary of all patients managed with naxitamab at HSJD is shown as supplementary material.
