# Supplementary material for: Clinical and Pathological Evidence of Anti-GD2 Immunotherapy Induced Differentiation in Relapsed/Refractory High-Risk Neuroblastoma
Source: Cancers (Basel). 2021 Mar 12;13(6):1264. doi: 10.3390/cancers13061264 (PMC7998131; doi:10.3390/cancers13061264)
Supplement: Supplementary file 1 [file cancers-13-01264-s001.pdf]

cancers

Supplementary Material: Clinical and Pathological Evidence of Anti-GD2 Immunotherapy Induced Differentiation in Relapsed/Refractory High-Risk Neuroblastoma

Jaume Mora [1,*], Alicia Castañeda [1], Maria Cecilia Colombo [2], Maite Gorostegui [1], Fernando Gomez [3], Salvador Mañe [4], Vicente Santa-Maria [1], Moira Garraus [1], Napoleon Macias [3], Sara Perez-Jaume [1], Oscar Muñoz [1], Juan Pablo Muñoz [1], Ignasi Barber [2] and Mariona Suñol [5]

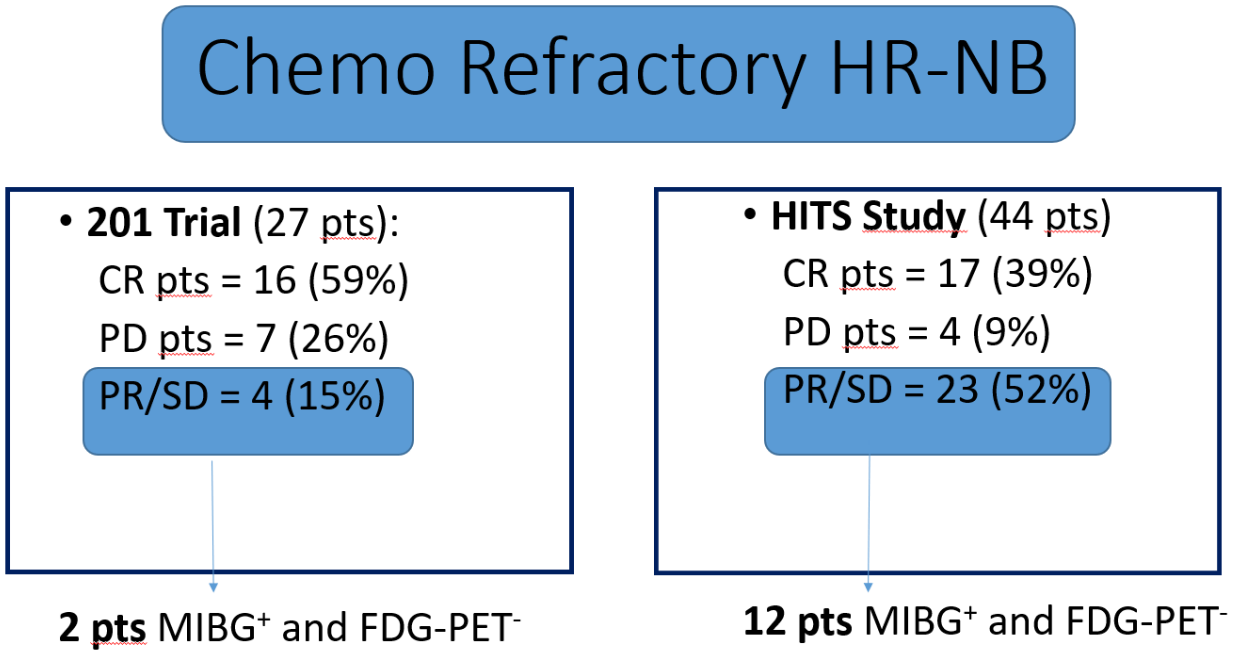

Figure S1. Histological picture of undifferentiated NB from patient #1 at diagnosis (before immunotherapy). A flow chart summary of all patients managed with naxitamab at HSJD is shown as supplementary material.
